# Macrophage Migration Inhibitory Factor Is a Molecular Determinant of the Anti-EGFR Monoclonal Antibody Cetuximab Resistance in Human Colorectal Cancer Cells

**DOI:** 10.3390/cancers11101430

**Published:** 2019-09-25

**Authors:** Rosita Russo, Nunzia Matrone, Valentina Belli, Davide Ciardiello, Mariangela Valletta, Sabrina Esposito, Paolo Vincenzo Pedone, Fortunato Ciardiello, Teresa Troiani, Angela Chambery

**Affiliations:** 1 Department of Environmental, Biological and Pharmaceutical Sciences and Technologies, Università degli studi della Campania “Luigi Vanvitelli”, 81100 Caserta, Italy; 2 Department of Precision Medicine, Università degli studi della Campania “Luigi Vanvitelli”, 80131 Naples, Italy

**Keywords:** cetuximab, colorectal cancer, drug resistance, MIF, mass spectrometry (MS)

## Abstract

Background: The clinical impact of the monoclonal antibody cetuximab targeting the EGFR in colorectal cancer (CRC) is widely recognized. Nevertheless, the onset of cetuximab resistance is a serious issue that limits the effectiveness of this drug in targeted therapies. Unraveling the molecular players involved in cancer resistance is the first step towards the identification of alternative signaling pathways that can be targeted to circumvent resistance mechanisms restoring the efficacy of therapeutic treatments in a tailored manner. Methods: By applying a nanoLC-MS/MS TMT isobaric labeling-based approach, we have delineated a molecular hallmark of cetuximab-resistance in CRC. Results: We identified macrophage migration inhibitory factor (MIF) as a molecular determinant capable of triggering cancer resistance in sensitive human CRC cells. Blocking the MIF axis in resistant cells by a selective MIF inhibitor restores cell sensitivity to cetuximab. The combined treatment with cetuximab and the MIF inhibitor further enhanced cell growth inhibition in CRC resistant cell lines with a synergistic effect depending on inhibition of key downstream effectors of the MAPK and AKT signaling pathways. Conclusions: Collectively, our results suggest the association of MIF signaling and its dysregulation to cetuximab drug resistance, paving the way to the development of personalized combination therapies targeting the MIF axis.

## 1. Introduction

Colorectal cancer (CRC) is one of the most frequently diagnosed cancers and one of the leading causes of cancer-related mortality worldwide [1]. Knowledge in cancer biology has allowed developing new drugs targeting specific pathways important for carcinogenesis, metastasis, proliferation and angiogenesis with a dramatic improvement of metastatic CRC (mCRC) patients’ outcomes [2]. In this scenario, epidermal growth factor receptor (EGFR) is an attractive target for anticancer therapy. 

Cetuximab and panitumumab, two monoclonal antibodies (mAbs) targeting the extracellular domain of the EGFR and inhibiting its activation by blocking its downstream intracellular signals (i.e., RAS-RAF-MEK-MAPK and PTEN-PIK3CA pathways) were the first targeted agents to enter the clinical setting improving the survival of mCRC patients [3,4,5]. In particular, both drugs are effective only in a subset of mCRC patients. In fact, mutations in *KRAS* and *NRAS* genes are found to predict primary resistance to anti-EGFR targeted therapies and are used in clinical practice to guide treatment decision [4,6]. In addition, a number of retrospective studies have provided evidence that primary resistance to EGFR inhibitors in colorectal cancer could be correlated to deregulation of other intracellular downstream effectors of EGFR, such as mutation in *BRAF* or *PIK3CA* genes, loss of *PTEN* expression, and amplification of *KRAS* [7,8,9,10]. However, even in patients who initially respond to anti-EGFR therapies, development of secondary resistance invariably occurs. The most common molecular mechanisms that are responsible for acquired resistance are genetic alterations of *KRAS*, *NRAS* and *BRAF* genes [11,12]. In the absence of alteration in *RAS* or its immediate downstream effectors, other mechanisms have been involved in the activation of the EGFR pathway. Genetic aberrations in receptor tyrosine kinase (RTK), such as human epidermal growth factor receptor 2 (*HER2*) and MET, have been shown to bypass EGFR signaling and activate the MAPK cascade and, therefore, to confer acquired resistance to anti-EGFR therapies [13,14,15,16,17]. Therefore, an understanding of these mechanisms is necessary to design effective therapies for patients to prevent and or overcome clinical resistance to cetuximab treatment.

Global genomic approaches based on microarray technology have been widely applied to identify genes and pathways at the basis of the molecular mechanisms of resistance and to establish new targets for rational design of new anticancer drugs [18,19,20].

In recent years, mass spectrometry (MS) has also become a powerful platform for the large-scale study of cellular and tissue proteomes, enabling the profiling at qualitative and quantitative level of proteins expressed by the genome, thus providing valuable information on molecular mechanisms regulating cellular events.

To date, studies focused on unravelling the expression signatures of cancer drug resistance at proteomic level are rapidly increasing, including the comparative analyses of complex protein mixtures from cell lines, tissue and body fluids. Several studies have been performed by using two-dimensional gel electrophoresis or LC-MS approaches mainly on breast cancer cell lines [21,22,23]. Recently, Wang and co-workers reported the identification of proteins potentially responsible for Adriamycin resistance in breast cancer by using an MS-based proteomic strategy [24]. Comparative proteome analyses also revealed differentially expressed proteins between tamoxifen-sensitive and tamoxifen-resistant breast cancer primary tumors [25]. A multi-omic characterization has been also performed on patient-derived xenograft tumors to determine intrinsic resistance mechanisms of triple-negative breast tumors to PI3K inhibition [26]. Furthermore, a shotgun proteomic platform has been employed to analyze stimulation and inhibition of EGFR by EGF and gefitinib and cetuximab EGFR inhibitors, respectively, on the A431 human epithelial carcinoma cell line as model system [27]. By this approach, a candidate “EGFR inhibition signature” was established and further validated in different EGFR sensitive (DiFi) and resistant (HCT116) cell lines. In addition, active kinase candidates in colorectal cancer cell lines sensitive (LIM1215 and DLD1) and resistant (HCT116 and HT29) to Cetuximab have been investigated by proteomic analysis [28].

In this work, the molecular determinants of cetuximab-resistance in colorectal cancer have been investigated. An advanced quantitative proteomic approach based on Tandem Mass Tag (TMT) isobaric labeling and nano-liquid chromatography coupled with high resolution tandem mass spectrometry (nanoLC MS/MS) has been utilized to identify and compare the expression levels of proteins in cetuximab-sensitive and resistant colon cancer cells. Our results provide a detailed characterization of the GEO cell line proteome relevant for EGFR signaling and associated to cetuximab-resistance, revealing a key role of Macrophage Migration Inhibitory Factor (MIF) in the mechanism of anti-EGFR sensitivity and resistance.

## 2. Results

### 2.1. Proteomic Analysis by High Resolution nanoLC−MS/MS on an In Vitro Preclinical Model of Colon Cancer Cells with Acquired Resistance to Cetuximab

To investigate the molecular determinants of acquired resistance to cetuximab in colon cancer, we took advantage of the previously characterized GEO human colon cancer cell model [15,16,29]. Despite the presence of a mutation in *KRAS* gene codon 12, GEO cancer cells are very sensitive to cetuximab treatment with an IC_50_ of 0.1 μg/mL (Figure S1) [15,29,30]. Interestingly, as previously described, prolonged treatments of GEO cells with increasing concentrations of cetuximab up to 6 months result in the loss of sensitivity to cetuximab at doses up to 20 μg/mL and the acquisition of resistance to the growth inhibitory effects of the drug [15,29,30] (Figure S1). The cetuximab-resistant cells (named GEO-CR) have been shown to maintain their properties in vitro in drug-free medium, thus representing a valuable preclinical model for elucidating mechanisms of cancer cell resistance [15,29,30].

**Figure 1:**
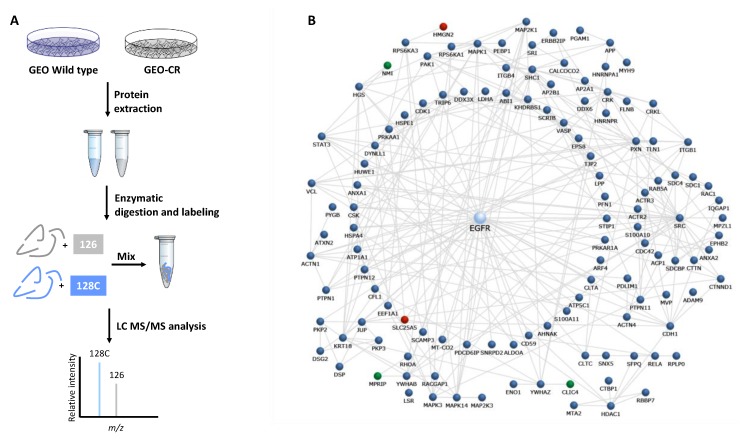
(**A**) Proteomic workflow for the investigation of molecular determinants of acquired resistance to cetuximab. For Tandem Mass Tag (TMT) isobaric labelling, proteins have been extracted from sensitive and cetuximab-resistant GEO cells, digested into peptides and labelled with TMT isobaric stable isotope tags. After mixing, in MS1, the peptides appear as a single precursor. When fragmented during MS2, in addition to the normal fragment ions, the reporter regions dissociate to produce ion signals which provide accurate quantitative information regarding the relative amount of the peptide in the samples. (**B**) Protein interaction network including a subset of proteins identified in GEO colon cancer cells mapping on EGFR1 pathway. Proteins mapping on EGFR1 pathway were identified in both sensitive and cetuximab-resistant GEO cell lines by performing an enrichment analysis against the human cancer and immune signaling pathways “NetPath” (Figure S3). These proteins were then mapped on the EGFR1 interaction network by the FunRich software. Up- and down-regulated proteins are colored in red and green, respectively. Proteins identified in both sensitive and cancer-resistant GEO cells by LC-MS/MS with no changes in their expression levels are reported in blue. For the network construction clusters with more than two nodes were only included. Interactions from outside the experimental dataset were excluded from the network. Molecules are named according to Funrich software.

In order to delineate a hallmark of GEO/GEO-CR colon cancer cells and identify candidate proteins responsible for their cancer resistance properties, a comparative proteomic analysis was performed in cetuximab-resistant GEO cells in comparison to parental sensitive cell line. We applied a quantitative proteomic approach based on TMT isobaric labeling and nano-liquid chromatography coupled with high resolution tandem mass spectrometry. The schematic representation of the experimental design is depicted in Figure 1A.

A high number of peptide groups (i.e., ~95,000) was used for protein identification, and out of these, about 80% were used as unique peptides for protein quantification attesting the high efficiency of peptide labeling. By MS/MS and database search, we identified and quantified 2380 non-reduntant proteins with more than one unique peptide in at least two out of three injections in both cell lines (Table S1). The enrichment analysis performed for the biological process Gene Ontology category (Figure S2A) revealed that a significant number of identified proteins were involved in processes related to signal transduction (38%), cell communication (32%) and cell growth and/or maintenance (14%). In addition, the most represented molecular function categories (Figure S2B) were those of protein serine/threonine kinase activity (7%), RNA (6.5%), cytoskeletal protein (6%) and calcium binding (6%) and receptor signaling complex scaffold activity (6%). Moreover, the clustering of these proteins performed according to cellular component classification revealed, besides the generic cytoplasm and nucleus categories, a significant enrichment of exosome (60%) and lysosome (38%) proteins in both sensitive and cetuximab-resistant GEO cell lines. More interestingly, the enrichment analysis performed on identified proteins in both sensitive and cetuximab-resistant GEO cell lines against the “NetPath” human cancer and immune signaling pathways database revealed a significant enrichment of proteins mapping on EGFR1 pathway (Figure S3). These proteins were extracted from the list of identified proteins and mapped on the EGFR1 interaction network, revealing the high-throughput potential of proteomic analysis in the characterization of GEO cells (Figure 1B).

**Figure 2:**
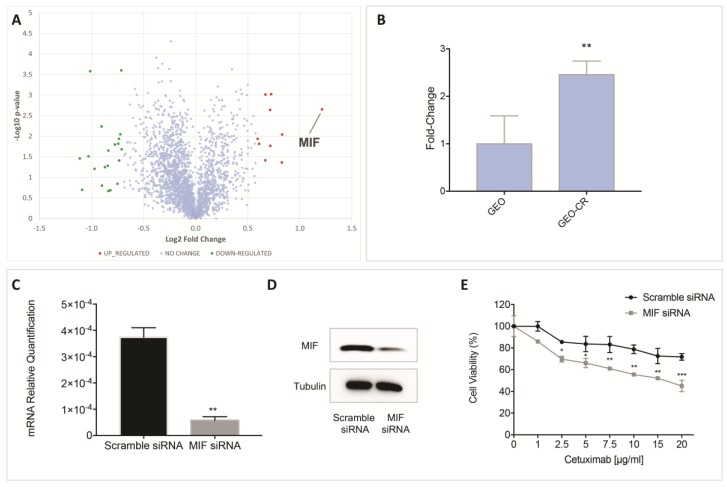
(**A**) Volcano plot obtained from TMT-based quantitative proteomics analysis of GEO-CR versus GEO cell lines. Each point represents the difference in expression (Log2 fold-change) between the cetuximab-resistant and sensitive GEO cell lines plotted against the −Log10 *p*-value. Identified proteins with no changes in their regulation level are reported in light blue. Up- and down-regulated proteins (fold change ≥ 1.5) are indicated in red and green, respectively. Differentially expressed proteins are reported in Table 1. (**B**) qRT-PCR analysis of MIF mRNA in GEO and GEO-CR cells. *bars*, standard deviation (SD); values are expressed as mean of four replicates (** *p* ≤ 0.01; Unpaired *t* test). (**C**) Targeted silencing of MIF gene expression by small interfering RNA (siRNA). Results of qRT-PCR detection of MIF expression in GEO-CR cells transfected with a scrambled siRNA used as negative control or siMIF; *bars*, standard deviation (SD); ** *p* < 0.01 as compared with the negative control. (**D**) Western blot showing gene silencing efficiency of siRNA sequences targeting MIF in GEO-CR cells. A scramble siRNA was used as negative control. (**E**) Cell viability was evaluated by MTT staining on GEO-CR cells transiently transfected with a scrambled siRNA used as negative control or siMIF following treatment for 72 h with cetuximab (range: 1–20 µg/mL). Inhibition of MIF by using a small RNA interference significantly re-sensitized resistant cells to cetuximab (* *p* ≤ 0.05; ** *p* ≤ 0.01; *** *p* ≤ 0.001; Two-way ANOVA).

Out of the identified proteins, a very small fraction (about 1%) was found to be differentially expressed (fold change ≥ 1.5) in cetuximab-resistant with respect to sensitive cell line (Figure 2A and Table 1). A specific subset of down-regulated proteins was mapped on the EGFR pathway, including the N-myc-interactor (NMPI), the chloride intracellular channel protein 4 (CLIC4) and the isoform 2 of myosin phosphatase Rho-interacting protein (MPRIP). In addition, two up-regulated proteins (i.e., the ADP/ATP traslocase 2, SLC25A5 and the non-histone chromosomal protein HMG-17, HMGN2) were also included in the EGFR protein–protein interaction network. Among identified proteins showing changes in their regulation levels, we focused our attention on the macrophage migration inhibitory factor (MIF), the differentially expressed protein with the highest up-regulation level. We therefore performed a cross-validation and confirmation of MS data by using qRT-PCR (Figure 2B) that confirmed MIF up-regulation in the cetuximab-resistant GEO-CR with respect to the sensitive GEO cells, suggesting a role for this factor in cetuximab-resistance.

Interestingly, inhibition of MIF by using a small RNA interference (siRNA) targeting the MIF gene significantly re-sensitized resistant cells to cetuximab (Figure 2C–E), prompting us to further investigate the involvement of MIF in cetuximab-resistance.

### 2.2. MIF Inhibition Affects Human Colon Cancer Cell Growth and Apoptosis

To determine the role of MIF in cetuximab-resistance, cell viability was evaluated on sensitive and resistant GEO cells treated with 4-iodo-6-phenylpyrimidine (4-IPP), a selective MIF inhibitor that covalently modifies MIF N-terminal proline [31].

**Figure 3:**
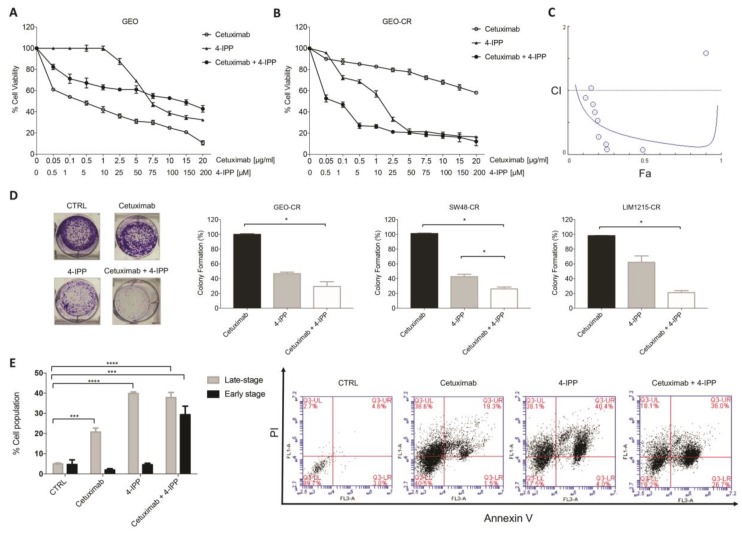
(**A**) MIF inhibition by the covalent inhibitor 4-IPP affects GEO (**A**) and GEO-CR (**B**) cell viability and apoptosis. Cell viability was evaluated by MTT staining following treatment for 72 h with cetuximab (range: 0.05–20 µg/mL), 4-IPP (range: 0.5–200 µM) or their combination at a fixed drug ratio of 1:10 (cetuximab:4-IPP). The IC_50_ values were determined by interpolation from the dose–response curves. Results represent the median of three separate experiments, each performed in triplicate. (**C**) The combination Index (CI) value was calculated according to the Chou and Talalay mathematical model for drug interactions using the CompuSyn software, and represent a quantitative measure of the degree of combination of cetuximab plus 4-IPP (i.e., CI < 0.8, synergism; 0.8 < CI < 1.2, additivity; CI > 1.2, antagonisms). (**D**) Clonogenic assay performed on GEO-CR, SW48-CR and LIM1215-CR cells after treatment for 72 h with cetuximab (2.5 µg/mL) and 4-IPP (25 µM) as single agents and in combination and then cultured for 14 days. Data are presented as means ± standard deviation (SD). * *p* ≤ 0.05 vs. control. (**E**) Bar chart showing the percentages of early and late apoptotic cells on GEO-CR cells following treatment with cetuximab (2.5 µg/mL), 4-IPP alone (25 µM) and their combination for 24 h and staining with annexin V/PI. The combination of Annexin V-FITC and propidium iodide allows for the distinction between early apoptotic cells (Annexin V-FITC positive) and late apoptotic and/or necrotic cells (Annexin V-FITC and propidium iodide positive). *bars*, standard deviation (SD); values are expressed as mean of three independent experiments; (*** *p* ≤ 0.001; **** *p* ≤ 0.0001; two-way ANOVA). Representative dual parametric dot plots of PI fluorescence (*Y*-axis) versus annexin V-FITC fluorescence (*X*-axis) are also reported.

As shown in Figure 3A,B, 4-IPP treatment at concentrations ranging from 0.5 to 200 μM for 72 h induced a dose-dependent cell growth inhibition in both sensitive and resistant GEO cell lines with an enhanced cell viability inhibition in GEO-CR cell lines with an IC_50_ value about four-fold higher in GEO-CR (Figure 3B) compared to GEO cells (Figure 3A). Interestingly, the combined treatment with cetuximab and 4-IPP further enhanced cell viability inhibition in GEO-CR cell lines with respect to single treatments (Figure 3B). Accordingly, a synergistic growth inhibitory effect was observed in GEO-CR with a combination Index (CI) value of 0.2, calculated according to Chou–Talalay method for drug interactions using CompuSyn software [32] (Figure 3C). These data suggest that the combined treatment of cetuximab-resistant cells with 4-IPP and cetuximab is able to restore the sensitivity of GEO-CR cells to cetuximab in a dose-dependent manner.

Interestingly, we confirmed that MIF blocking by 4-IPP also inhibits cell viability on two previously described cetuximab-resistant CRC cell lines [15,16,30]: LIM1215-CR (Figure S4A) and SW48-CR (Figure S4B). Moreover, as observed for GEO-CR cells, the combined treatment with cetuximab and 4-IPP also had significant synergistic effects on cell viability inhibition (Figure S4C,D, CI values of 0.6 and 0.2 for LIM1215-CR and SW48-CR cells, respectively).

**Figure 4:**
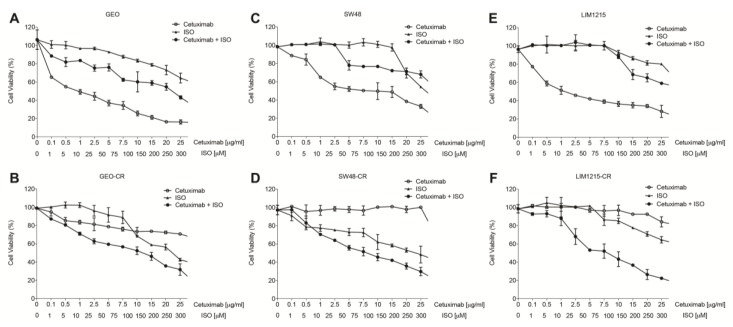
(**A**) MIF inhibition by the non-covalent inhibitor ISO-1 affects GEO (**A**) and GEO-CR (**B**), SW48 (**C**) and SW48-CR (**D**), LIM1215 (**E**) and LIM1215-CR (**F**) cell viability. Cell viability was evaluated by MTT staining following treatment for 72 h with cetuximab (range: 0.1–25 µg/mL), ISO-1 (range: 1–300 µM) or their combination at a fixed drug ratio of 1:10 (cetuximab:ISO-1).

These findings were then confirmed by using the non-covalent ISO-1 MIF inhibitor. As expected, based on their mechanisms of actions, the effects were more evident at higher inhibitor concentrations of the non-covalent with respect to the covalent inhibitor. Nevertheless, ISO-1 and cetuximab clearly display synergistic activity when used in combination on cetuximab-resistant GEO-CR, LIM1215-CR and SW-48-CR cell lines in a dose-dependent manner (Figure 4).

To assess the effects of single and combined treatments at longer times, clonogenic survival assays were then performed on GEO-CR cells. In agreement with previous results, the combination of cetuximab and 4-IPP significantly enhanced clonogenic growth inhibition compared to either single agent (Figure 3D). This effect was also observed on LIM1215-CR and SW48-CR cetuximab-resistant cell lines (Figure 3D).

Once verified that treatments of GEO-CR cells with single and combined agents did not affected MIF expression levels (Figure S5), we investigated cell cycle distribution in GEO-CR cells by PI staining and DNA content evaluation by flow cytometry to probe whether cell cycle arrest contributed to cell viability inhibition by 4-IPP alone or in combination with cetuximab.

As shown in Figure S6, no significant cell cycle effects were observed after all treatments whereas, according to the synergistic growth inhibitory effect of cetuximab and 4-IPP, we found a significant induction of apoptosis in GEO-CR cells following the combined treatment with the two drugs (Figure 3E). In particular, the AnnexinV/PI double staining revealed that while a significant increase of late apoptotic and/or necrotic cells (Annexin V and PI positive) occurred following both single and combined treatments, a significant increase of early apoptotic cells (Annexin V positive) was only observed following the combined treatment (Figure 3E), further confirming the synergistic potential of the two agents in inhibiting cell growth by inducing apoptosis. Accordingly, the combination treatment did also significantly increase apoptosis in both LIM1215-CR and SW48-CR cells compared to their respective controls and either single agents (Figure S7).

**Figure 5:**
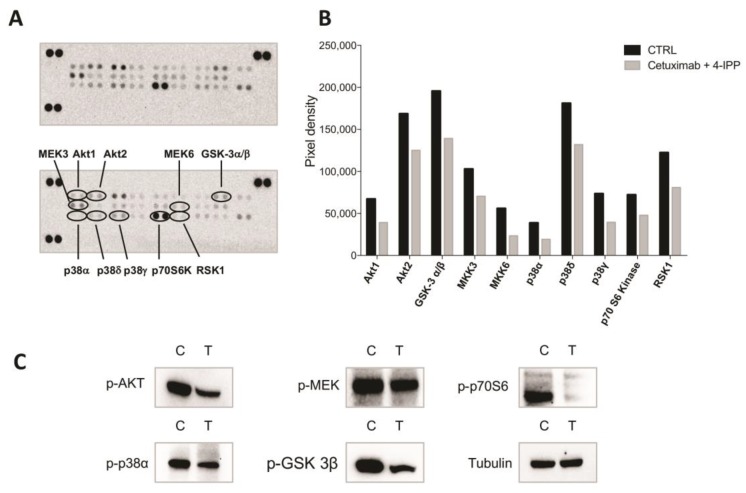
Combined treatment with cetuximab and 4-IPP inhibits the MAPK and AKT signaling pathways in resistant GEO cells. (**A**) 300 μg of protein lysates from GEO-CR cell line untreated (upper panel) and treated with cetuximab (2.5 μg/mL) and 4IPP (25 μM) (lower panel) were analyzed by human phospho-kinase array evaluating the phosphorylation of the following proteins: Akt1, Akt2, Akt3, Akt pan, CREB, ERK1, ERK2, GSK-3α/β, GSK-3β, HSP27, JNK1, JNK2, JNK3, JNK pan, MEK3, MEK6, MSK2, p38α, p38β, p38δ, p38γ, p53, p70 S6 Kinase, RSK1, RSK2, TOR. Signals of downregulated kinases (decrease ≥ 30%) in response to combined treatment are indicated. (**B**) Bar graph showing quantification of normalized mean spot pixel densities of untreated (black) versus treated (grey) cells for downregulated (decrease ≥ 30%) phosphorylated kinases. (**C**) Western blot analysis of AKT, MEK, p70 S6, p38α, GSK-3β phosphorylation in GEO-CR cell line untreated (C) and treated (T) with cetuximab (2.5 μg/mL) and 4IPP (25 μM).

In order to investigate the downstream signals of the cetuximab and 4-IPP combined treatment, we evaluated their effects on the activation/inhibition of key EGFR signaling effectors on GEO-CR cells by using a human phospho-MAP kinase array (Figure 5A,B). We observed a downregulation of the phosphorylation levels of Akt1/2, GSK-3 α/β, MEK3/6, p38 α /γ/δ, p70S6 kinase and RSK1 (Figure 5A,B), supporting the hypothesis that the synergic treatment overcomes cetuximab-resistance by inhibiting key downstream effectors of the MAPK and AKT signaling pathways. These results were further confirmed by western blot analyses for p-AKT, p-MEK, p-GSK-3 β, p-p38 α, p-p70S6 (Figure 5C).

### 2.3. MIF Is Able to Trigger Cetuximab-Resistance in Sensitive Colon Cancer Cells

**Figure 6:**
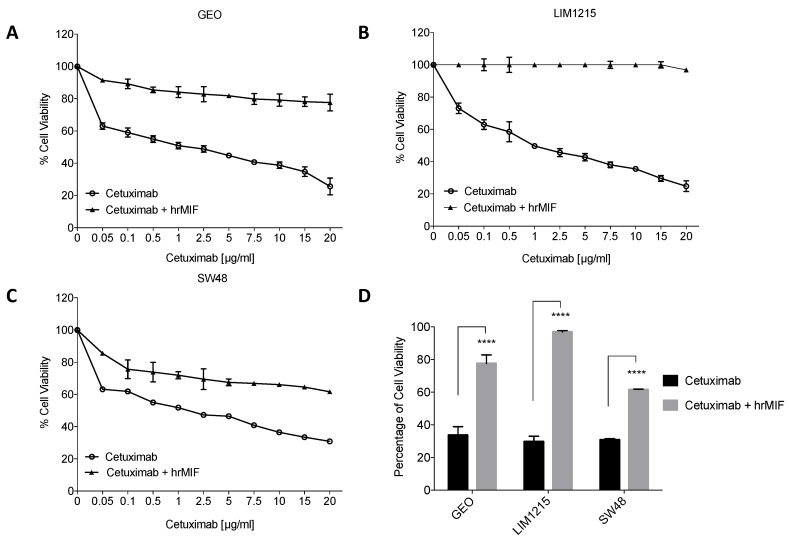
MIF triggers cetuximab-resistance in sensitive colon cancer cells. (**A**–**C**) Cell viability was evaluated by MTT staining on GEO (**A**), LIM1215 (**B**) and SW48 (**C**) cells following treatment for 72 h with increasing concentrations of cetuximab (range 0.05–20 µg/mL), alone and in combination with human recombinant MIF (hrMIF, 100 ng/mL). Results represent the median of three separate experiments, each performed in triplicate. (**D**) Graph bar showing the cell viability percentage of each cell line treated with cetuximab alone (20 µg/mL) and in combination with hrMIF (100 ng/mL). *bars*, standard deviation (SD); cetuximab versus cetuximab plus hrMIF; **** *p* ≤ 0.0001.

To evaluate the capability of MIF of modulating resistance to cetuximab in CRC cells, cell proliferation rate was evaluated on sensitive GEO cells treated with cetuximab in the presence and absence of human recombinant MIF (hrMIF) at 100 ng/mL for 24 h. Notably, while no changes in the expression levels of endogenous MIF were revealed by western blot analyses following either single and combined treatment with hrMIF and cetuximab (Figure S8), treatment with exogenous MIF triggers cetuximab-resistance in sensitive GEO cells (Figure 6A,D). This effect was further confirmed in two additional sensitive colon cancer cell lines (i.e., LIM1215 and SW48 cells) (Figure 6B–D). The significant reduction of cetuximab-induced cell growth inhibition following hrMIF treatment strongly supports its involvement in the mechanisms of the acquired resistance to cetuximab in colon cancer.

## 3. Discussion

Dysregulated epidermal growth factor receptor (EGFR) expression and intracellular signaling play a crucial role in the etiology of many human cancers [33]. Indeed, activation of the EGFR autocrine growth pathway is a common mechanism for cancer cell proliferation, adhesion, migration and invasion in different types of epithelial cancers including CRC. EGFR overexpression in about 70–80% of metastatic CRC has been also associated with poor prognosis and with the development of resistance to anticancer treatments [20,34,35,36]. Therefore, therapeutic strategies targeting EGFR have been developed in the last decade to inhibit EGFR activation and its associated downstream signaling pathways [37]. In this context, monoclonal antibodies (mAbs) represent an exciting and rapidly developing class of drugs for their undoubted advantages in terms of specificity [37].

Cetuximab (Erbitux) is a human/murine chimeric mAb that selectively binds to the extracellular domain III of EGFR. This interaction results in the competitive inhibition of endogenous ligand binding with the consequent blockade of EGFR signaling [34,37,38,39]. Antitumor activity of cetuximab has been well established against a variety of cancer cell lines in preclinical studies [34]. Nevertheless, intrinsic and acquired cetuximab resistance represent a serious issue that limits the effectiveness and restricts the use of this therapeutic drug in targeted therapies [34]. To date, different mechanisms of resistance to anti-EGFR drugs in CRC have been described including reduced antibody-receptor interaction, activation of parallel subsidiary pathways, constitutive activation of EGFR effector molecules, reactivation of pro-angiogenic factors, dysregulation of EGFR internalization and degradation [20,35,36]. The complexity of the resistance phenomenon requires the setting up of approaches based on preclinical models and high-throughput methodologies for understanding the molecular mechanisms responsible for resistance and the identification of additional key signaling pathways that can be targeted to enhance mAbs activity.

The advent of pangenomic and transcriptomic approaches provides unprecedented opportunities to unravel mechanisms of drug resistance through the assessment of novel predictive biomarkers of sensitivity and resistance to anti-EGFR mAbs. In this framework, somatic KRAS mutations have been identified as one of the most predictive markers of response to cetuximab in CRC, directing these targeted therapies to patients with wild-type KRAS [12,20,36]. Nevertheless, a significant number of patients with no detectable mutations in KRAS failed to respond to cetuximab treatments whereas patients harboring KRAS mutations are sensitive to cetuximab-based therapies, suggesting the occurrence of additional mechanisms at the basis of cetuximab responsiveness [40].

In the effort to improve overall understanding of molecular determinants of cetuximab resistance in CRC and to identify potential subsidiary pathways to be targeted to overcome resistance, we applied an advanced quantitative proteomic approach based on TMT isobaric labeling coupled to nanoLC MS/MS. As preclinical model system of human colon cancer, we used the established sensitive and cetuximab-resistant GEO cell lines (GEO and GEO-CR, respectively), proved to be a valuable tool for elucidating mechanisms of acquired cancer cell resistance since parental cells are very sensitive to cetuximab treatment despite the presence of a mutation in KRAS [15]. In particular, it has been reported that cetuximab treatment of sensitive GEO cells induced cell growth inhibition and apoptosis by reducing MAPK and AKT phosphorylation [15]. On the contrary, in resistant GEO-CR cells, these pathways were not blocked by cetuximab-treatment suggesting the occurrence of mechanisms bypassing EGFR inhibition and sustaining MAPK activation [15].

By performing a proteome-wide profiling of GEO cell lines, we mapped a subset of proteins relevant for EGFR signaling including a subset of proteins previously reported to be involved in mechanisms of cancer resistance. Among these, the ADP/ATP translocase 2 (SLC25A5, also known as Adenine nucleotide translocase-2, ANT2) has been previously reported to be overexpressed in CRC and other cancer models [41,42]. The overexpression of this oncogenic mitochondrial membrane–associated protein has been also suggested to contribute to resistance to the EGFR tyrosine kinase inhibitor gefitinib in NSCLC [43].

In addition, we identified Macrophage Migration Inhibitory Factor (MIF) as a molecular determinant of the anti-EGFR cetuximab resistance in human colorectal cancer cells.

The strong up-regulation of MIF in cetuximab-resistant GEO cells prompted us to investigate its involvement in triggering resistance. Our results provide new insights on the MIF capability of promoting cetuximab resistance in sensitive colon cancer cells.

We indeed find that MIF is able to trigger cetuximab-resistance in GEO as well as two other sensitive CRC cells (i.e LIM1215 and SW48). Interestingly, MIF inhibition by siRNA re-sensitized cetuximab-resistant GEO cells to cetuximab and MIF blocking by the covalent 4-IPP and the non-covalent ISO-1 MIF inhibitors suppress cell growth in both sensitive and resistant GEO cell lines. Of note, the combined treatment with cetuximab and 4-IPP or ISO-1 further enhanced proliferation inhibition in the resistant cell line with a synergistic growth inhibitory effect paralleled by a significant induction of apoptosis in GEO-CR cells following the combined treatment with the two drugs. We also show that combination with cetuximab and 4-IPP effectively inhibits key downstream effectors of the MAPK and AKT signaling pathways, suggesting that MIF-induced MAPK and AKT activation is involved in the mechanism of intrinsic resistance to cetuximab.

Our findings agree with recent studies demonstrating that MIF displays pro-tumorigenic activity and has a causal role in the pathogenesis of inflammatory diseases and cancer. Besides its pivotal role as a regulator of innate immunity, an increasing number of functions are being described for this pleiotropic cytokine/growth factor [44,45]. Among these, MIF possesses unusual tautomerase and oxidoreductase catalytic activities with still unclear pathophysiological relevance [44,45,46]. In addition, MIF is able to promote inflammatory processes acting as a chemokine and exerting its chemoattractant activity as a non-cognate ligand of the chemokine receptors CXCR2 and CXCR4 [47]. Reportedly, both exogenous and endogenous MIF expression promote cellular proliferation and inhibition of apoptosis through various mechanisms including a direct interaction with p53 that blocks its translocation from the cytoplasm to the nucleus and, at extracellular level, the interaction with the cell surface antigen CD74 [48]. The binding of MIF to CD74 triggers its dimerization with CD44 and the activation of MAPK signaling causing an inhibition of apoptosis and promotion of cell division [48].

Increasing evidence is emerging on the pivotal role of MIF multiple functions in the onset and maintenance of carcinogenesis in various neoplastic diseases, including lung [31], bladder [49] and breast [50] cancer. Over the last decade, multiple roles for MIF have been also reported in promoting colon cancer development and progression [46,51,52,53]. Furthermore, significant evidence is emerging linking MIF overexpression with increased invasion and metastasis of colorectal carcinoma supporting a correlation of expression levels of this cytokine with a more aggressive phenotype [46]. Accordingly, it has been reported that colon cancers and colon cancer cell lines also abundantly express MIF receptors CXCR2 [54] and CXCR4 [46,52]. Interestingly, drug-resistant HT-29 colon carcinoma cells selected after chronic treatment with different drugs (i.e., 5-fluorouracil, methotrexate, doxorubicin or oxaliplatin) also exhibited high expression of CXCR4. In these drug-resistant colon cancer cell populations, MIF was identified as the critical autocrine CXCR4 ligand promoting the invasive potential [52]. Although MIF is only one of multiple ligands of these receptors, these studies highlight the high therapeutic potential of MIF inhibition in combination therapies to potentially overcome intrinsic or acquired resistance to chemotherapeutic agents associated with the activation of MIF downstream effectors.

## 4. Materials and Methods

### 4.1. Materials

Cetuximab, an anti-EGFR human mouse chimeric mAb was kindly provided by Merck Serono (Rome, Italy), and it was ready to use. 4-IPP, a MIF Antagonist III and the non-covalent ISO-1 MIF inhibitor were purchased from Calbiochem and Millipore, respectively, and were dissolved in sterile dimethylsulfoxide (DMSO) at 10 mM working solution and stored in aliquots at −20 °C. All chemicals, tosyl phenylalanyl chloromethyl ketone (TPCK)- treated trypsin were from Sigma-Aldrich (Milan, Italy), unless otherwise stated. Human recombinant MIF (SRP3321) was purchased from Sigma (Milan, Italy). Acetonitrile (CH_3_CN), formic acid (FA) and water LC- MS grade were from Fisher Scientific Italia.

### 4.2. Human Cancer Cell Lines

The human colon cancer cell line GEO was kindly provided by Dr. N. Normanno (National Cancer Institute, Naples, Italy). The human LIM1215 and SW48 colon cancer cell lines were purchased from the American Type Culture Collection (ATCC). The GEO-CR, SW48-CR and LIM1215-CR cell lines were established as previously described [15,16,29,55]. The LIM1215, LIM1215-CR, SW48 and SW48-CR cancer cells were grown in RPMI-1460 medium (Sigma-Aldrich, Milan, Italy), whereas GEO and GEO CR cells were grown in McCoy’s 5A Modified Medium (Sigma-Aldrich, Milan, Italy) supplemented with 10% fetal bovine serum (FBS; Gibco, Thermo Fisher Scientific, Rodano MI, Italy), 1% penicillin/streptomycin (Sigma-Aldrich). All cell lines were maintained at 37 °C in a humidified atmosphere with 5% carbon dioxide (CO_2_). All cell lines were routinely screened for the presence of Mycoplasma using PlasmoTest-detection kit (InvivoGen, Toulouse, France).

### 4.3. Proliferation Assay

Colorectal cancer cells lines were seeded into 24-well plates at the density of 1 × 10^4^ cells/well and were exposed to different concentrations of cetuximab (range, 0.05–20 µg/mL) alone or in combination with 4-IPP (range, 0.5–200 µM) for 72 h. Cetuximab treatment on sensitive GEO, LIM1215 and SW48 cells was also performed in combination with human recombinant MIF (hrMIF, 100 ng/mL). Cell viability was measured with the 3-(4,5-dimethylthiazol-2-yl)-2,5-diphenyl-tetrazolium bromide (MTT) solution (final concentration, 5 mg/mL, Sigma-Aldrich). The MTT solution was removed and formazan crystals were extracted with isopropanol supplemented with 1% of HCl (200 µL/well). The 24-well plates were stirred up for 10 min and, subsequently, 100 μL of solution were transferred into 96-well. Absorbance of the formazan’s solution in isopropanol-HCl was measured spectrophotometrically at a wavelength of 550 nm. The IC_50_ value was determined by interpolation from the dose–response curves. Results represent the median of three separate experiments, each performed in triplicate. Results of the combination treatment were analyzed according to the method of Chou and Talalay by using the CompuSyn software program (Biosoft, Cambridge, UK).

Treatments with the non-covalent MIF inhibitor ISO-1 were performed on sensitive and resistant GEO, SW48 and LIM1215 colorectal cancer cells seeded into 48-well plates at the density of 6 × 10^3^ cells/well and exposed to different concentrations of cetuximab (range, 0.1–25 μg/mL) alone or in combination with ISO-1 (range, 1–300 μM) for 72 h. The growth inhibition was assessed by the MTT assay as described above.

### 4.4. RNA Extraction and qRT-PCR

Total RNA was prepared using TRIsure reagent (BioLine, Randolph, MA, USA) and reverse-transcribed into cDNA by SensiFast reverse transcriptase (BioLine), according to the manufacturer’s instructions. qRT-PCR analysis was performed using the following primers: MIF Fw: 5′-CCCGGACAGGGTCTACATCA-3′; MIF Rv: 5′-GGAGTTGTTCCAGCCCACAT-3′; 18S Fw: 5′-GGCGACGACCCATTCGAAC-3′; 18S Rv: 5′-AGGCACGGCGACTACCATC-3′; Amplification was conducted using the SYBR Green PCR Master Mix (Applied Biosystems, Foster City, CA, USA). All samples were run in duplicate using a Quant studio 7 Flex (Applied Biosystem). 18S ribosomal RNA (18S rRNA) was used as reference genes to normalize qRT-PCR data. To calculate MIF fold change, the 2^−ΔΔCt^ method was used. Values are expressed as mean of four replicates.

### 4.5. RNA Interference

A small RNA interference (siRNA) targeting the MIF gene (siRNA MIF, Ambion-Fisher scientific Italia, Rodano MI, Italy) was used for silencing the target gene. A scrambled siRNA was used as negative control. All siRNAs were transiently transfected in GEO-CR cell lines with Lipofectamine 300 (Invitrogen, Carlsbad, CA, USA), according to the manufacturer’s instructions. MIF expression was analyzed by qRT-PCR and western blot analysis as described in paragraphs 4.4 and 4.10, respectively.

### 4.6. Colony Formation Assay

Colorectal cancer cells were seeded into 6-well plates (3 × 10^3^cells/well) and treated with cetuximab (2.5 µg/mL) and 4-IPP (25 µM) as single agents and in combination for 72 h and then followed for an additional 14 days. Afterwards, cells were fixed with 4% paraformaldehyde and stained with crystal violet. The number of colonies were calculated after cell lysis and quantified spectrophotometrically at 595 nm. Two independent experiments were performed and data were presented as mean ± standard deviation (SD).

### 4.7. Cell Cycle Assay

Colorectal cancer cells were seeded in 6-well plates and treated with cetuximab (2.5 μg/mL) and 4-IPP (25 μM), as single agents and in combination for 24 h. Cells were harvested and suspended in different buffers included in BD Cycletest Kit, according to the manufacturer’s instructions (BD, Bioscience, San Jose, CA, USA). FACS analyses were performed by using the BD Accuri C6 Flow Cytometer (BD Biosciences). For each sample, 2 × 10^4^ events were acquired. Data were analyzed using GraphPad Prism software (GraphPad Software, San Diego, CA, USA).

### 4.8. Apoptosis Assay

The GEO-CR, LIM1215-CR, SW48-CR cells were seeded in 6-well plates, treated with cetuximab (2.5 µg/mL), 4-IPP (25 µM) or their combination for 24 h. Cells were harvested with trypsin, washed with PBS and suspended in binding buffer (Invitrogen, Carlsbad, CA, USA). Subsequently 5 µL of Annexin V-FITC (Invitrogen) and 5 µL of propidium iodide (Invitrogen) were added into cells suspension and incubated at room temperature in the dark for 30 min. The analysis was performed by using the BD Accuri C6 Flow Cytometer (BD Biosciences, San Jose, CA, USA). Results represent the median of three separate experiments, each performed in duplicate. Histogram analysis was performed by using the GraphPad Prism software.

### 4.9. Phospho-MAPK Profiling Array

Phospho-proteome profiler array has been performed by following manufacturer’s protocol (Human Phospho-MAPK Array Kit, R&D Systems, Minneapolis, MN, USA). Briefly, GEO-CR cancer cells were seeded in 6-well plates, treated with cetuximab (2.5 μg/mL) and 4-IPP (25 μM) for 24 h, rinsed with PBS and solubilized with Lysis Buffer 6 (provided in Human Phospo-MAPK Array) at 1 × 10^7^ cells/mL. Samples were rocked for 30 min at 4 °C, centrifuged for 5 min at 14,000× *g* at 4 °C and the supernatants were collected and subjected to protein content determination by using the BioRad Reagent (BioRad, Milan, Italy). Protein lysates (300 μg) were incubated overnight with human phospho-kinase array in order to analyze the phosphorylation profile of the following 26 kinases: Akt1, Akt2, Akt3, Akt pan, CREB, ERK1, ERK2, GSK-3α/β, GSK-3β, HSP27, JNK1, JNK2, JNK3, JNK pan, MEK3, MEK6, MSK2, p38α, p38β, p38δ, p38γ, p53, p70 S6 Kinase, RSK1, RSK2, TOR. The levels of phosphorylated proteins were analyzed by quantification of the pixel density of each spot on the array by using the ImageLab software (BioRad), according to the manufacturer’s recommendations.

### 4.10. Western Blot Analysis

GEO-CR cell lines were treated with cetuximab (2.5 μg/mL) and 4-IPP (25 μM) for 24 h. Equal amounts of total proteins (25 μg) were incubated with the following primary polyclonal antibodies: p-AKT, p-MEK, p-p70S6, p-p38α and p-GSK-3β purchased from Cell Signaling and anti-MIF antibody (1:500; Sigma). The monoclonal anti-α-tubulin antibody (Sigma-Aldrich) was used as loading control antibody. After incubation with the secondary antibody, membranes were developed using an enhanced chemi-luminescence (ECL) detection system (BioRad). Densitometry readings/intensity ratios normalized with tubulin along with the whole blot showing all the bands with all molecular weight markers of Western blot sections of Figure 2D and Figure 5C are reported in the Figures S9–S11.

### 4.11. Sample Preparation for Proteomic Analyses

For proteomic analysis, sensitive (GEO) and cetuximab-resistant (GEO-CR), human colorectal cancer GEO cell lines were lysed in ice-cold lysis buffer (100 mM Tetraethylammonium bicarbonate TEAB, SDS 1%) and disrupted by two cycles of sonication at a 20% amplitude for 30 sec on ice. Lysates were cleared by centrifugation at 16,000× *g* for 15 min at 4 °C. Supernatants were transferred into new tubes and treated with 1 Unit of RQ1 DNase (Promega, Milan, Italy) for 1 h at room temperature. Protein concentration was determined by using the Pierce BCA Protein assay kit (Thermo Scientific, Rodano MI, Italy). For each condition, equal amounts of proteins (100 µg in 100 µL of 100 mM TEAB) were reduced with 10 mM Tris-(2-carboxyethyl)-phosphine (TCEP) for 1 h at 55 °C and alkylated with 18 mM iodoacetamide by incubating samples for 30 min at room temperature in the dark. Proteins were then precipitated overnight by adding six volumes of pre-chilled acetone. Following centrifugation at 8,000× *g* for 10 min at 4 °C, protein pellets were resuspended in 100 µL of 100 mM TEAB and digested overnight with MS grade trypsin (Thermo Scientific, Rodano MI, Italy) at an enzyme/substrate ratio of 1:40 at 37 °C. Resulting peptide mixtures were chemically labeled with the TMT isobaric tags as previously reported [56] using the 128C and 126 tags for the GEO and GEO-CR samples, respectively. Briefly, 0.8 mg of TMT reagents in 41 µl of anhydrous acetonitrile were added to each sample. The reaction proceeded for 1 h and then was quenched for 15 min with hydroxylamine to a final concentration of 0.3%. The two samples were then mixed at equal amounts and diluted in 0.1% TFA/2% CH_3_CN to a final concentration of 0.5 µg/µL for LC-MS analyses.

### 4.12. High Resolution nanoLC−Tandem Mass Spectrometry

Aliquots of TMT labeled samples (2.5 µg) were analyzed in triplicate by high resolution nanoLC−Tandem Mass Spectrometry using a Q-Exactive Orbitrap mass spectrometer equipped with an EASY-Spray nano-electrospray ion source (Thermo Fisher Scientific, Rodano MI, Italy) and coupled to a Thermo Scientific Dionex UltiMate 3000RSLC nano system (Thermo Fisher Scientific). Solvent composition was 0.1% formic acid in water (solvent A) and 0.1% formic acid in acetonitrile (solvent B). Peptides were loaded on a trapping PepMap™100 μCartridge Column C18 (300 μm × 0.5 cm, 5 μm, 100 Å) and desalted with solvent A for 3 min at a flow rate of 10 μL/min. After trapping, eluted peptides were separated on an EASY-Spray analytical column (50 cm × 75 μm ID PepMap RSLC C18, 3 μm, 100 Angstrom), heated at 35 °C at a flow rate of 300 nL/min applying the following gradient: 5% B for 3 min, from 5% to 27.5% B in 222 min, from 27.5% to 40% B in 10 min, from 40% to 95% B in 1 min. Washing (95% B for 4 min) and re-equilibration (5% B for 24 min) steps were always included at the end of the gradient. Eluting peptides were analyzed on the Q-Exactive mass spectrometer operating in positive polarity mode with capillary temperature of 280 °C and a potential of 1.9 kV applied to the capillary probe [57]. Full MS survey scan resolution was set to 70,000 with an automatic gain control (AGC) target value of 3 × 10^6^ for a scan range of 375−1500 m/z and maximum ion injection time (IT) of 60 ms. The mass (m/z) 445.12003 was used as lock mass. A data-dependent top 12 method was operated during which high-energy collisional dissociation (HCD) spectra were obtained at 35,000 MS2 resolution with AGC target of 1 × 10^5^ for a scan range of 200−2000 m/z, maximum IT of 120 ms, 1.6 m/z isolation width and normalized collisional energy (NCE) of 32. Precursor ions targeted for HCD were dynamically excluded for 30 s. Full scans and Orbitrap MS/MS scans were acquired in profile mode, whereas ion trap mass spectra were acquired in centroid mode. Charge state recognition was enabled by excluding unassigned and 1, 7, 8, >8 charged states. All data were acquired with the Xcalibur 3.1 software (Thermo-Fisher Scientific).

### 4.13. Protein Identification and Quantitation

For data processing, the acquired raw files were analyzed with the Thermo Scientific Proteome Discoverer 2.1 software (Thermo Fisher Scientific) using the SEQUEST HT search engine. The HCD MS/MS spectra were searched against the *Homo sapiens* database (version 2015-11-11, number of entries 42,084 sequences) assuming trypsin (Full) as digestion enzyme and two allowed number of missed cleavage sites. Mass tolerances were set to 10 ppm and 0.02 Da for precursor and fragment ions, respectively. Oxidation of methionine (+15.995 Da) was set as dynamic modification. Carbamidomethylation of cysteine (+57.021 Da) and the TMT label on lysines and the N-terminus (229.1629) were set as static modifications. False discovery rates (FDRs) for peptide spectral matches (PSMs) were calculated and filtered using the Percolator node in Proteome Discoverer that was run with the following settings: Maximum Delta Cn 0.05, a strict target FDR of 0.01, a relaxed target FDR of 0.05 and validation based on *q*-value. Protein identifications were accepted when the protein FDR was below 1% and when present in at least two out of three replicate injections with at least two peptides.

### 4.14. Bioinformatic Analyses

Functional enrichment based on gene ontology categories and interaction network analyses of proteins identified by LC-MS/MS were performed by the FunRich open access software (http://funrich.org/index.html). An enrichment analysis of significantly over-represented biological pathways was also performed against the manually curated NetPath human cancer and immune signaling pathways database by using the Pathway Analysis tool of the InnateDB integrated analysis platform (http://www.innatedb.com/index.jsp).

## 5. Conclusions

Taken together, our results attest an upregulation of MIF expression in cetuximab-resistant colon cancer cell associating, for the first time, MIF dysregulation to cetuximab drug resistance. This finding strongly supports previous studies and further delineates an unexpected complexity of MIF involvement in cancer resistance.

Indeed, we might envisage a scenario in which inhibition of the EGFR pathway leads to activation of MIF-related parallel signaling pathways decreasing the effectiveness of single-MEK targeted therapies. In line with our findings, Cheon and co-workers recently demonstrated for other KRAS mutant colorectal cancer cells a bypass mechanism of refametinib resistance to MEK inhibition involving the activation of STAT3 and MAPK triggered by MIF overexpression [53]. According to our data on the synergistic effect of the cetuximab/4-IPP combined treatment, combination with refametinib/4-IPP significantly induced regression of tumor growth while this effect was not observed following single treatments [53].

While more extensive studies are needed to expand our knowledge on mechanisms behind the impaired MIF-driven signaling in cancer resistance, we believe that our results pave the way to further investigations aiming at exploring rational combination therapies targeting MIF axis to overcome drug resistance.

## Figures and Tables

**Table 1:** Proteins differentially regulated identified in cetuximab-resistant GEO cell lines by high-resolution LC-MS/MS. A total of 26 out of 2380 proteins were found to be differentially expressed (0.6 ≥ FC ≥ 1.5) in cetuximab-resistant with respect to sensitive GEO cell lines.

Accession	Description	Gene	GEO_CR/GEO	Coverage	# PSMs	# Peptides	MW [kDa]
P14174	Macrophage Migration inhibitory factor	*MIF*	2.3	26.1	10	2	12.5
Q99439	Calponin-2	*CNN2*	1.8	40.5	30	7	33.7
Q7LBR1	Charged multivesicular body protein 1b	*CHMP1B*	1.8	7.5	4	2	22.1
Q9UEE9-1	Craniofacial development protein 1	*CFDP1*	1.7	6.7	4	2	33.6
P84101-1	Small EDRK-rich factor 2	*SERF2*	1.6	40.7	6	3	6.9
P05204	Non-histone chromosomal protein HMG-17	*HMGN2*	1.6	30.0	23	3	9.4
P05141	ADP/ATP translocase 2	*SLC25A5*	1.6	42.3	184	5	32.8
P56381	ATP synthase subunit epsilon, mitochondrial	*ATPSF1E*	1.6	29.4	5	2	5.8
P09234	U1 small nuclear ribonucleoprotein C	*SNRPC*	1.5	13.2	5	2	17.4
Q01844-5	Isoform 5 of RNA-binding protein EWS	*EWSR1*	1.5	11.5	22	6	68.9
Q5VW32	BRO1 domain-containing protein BROX	*BROX*	0.6	11.7	10	4	46.4
O14493	Claudin-4	*CLDN4*	0.6	16.7	10	2	22.1
Q99795	Cell surface A33 antigen	*GPA33*	0.6	33.9	59	10	35.6
O94901-9	Isoform 9 of SUN domain-containing protein 1	*SUN1*	0.6	4.7	5	3	101.9
P21912	Succinate dehydrogenase [ubiquinone] iron-sulfur subunit	*SDHB*	0.6	14.3	6	4	31.6
Q13287	N-myc-interactor	*NMI*	0.6	9.4	4	3	35.0
Q9UBC2-2	Isoform 2 of Epidermal growth factor receptor substrate 15-like 1	*EPSISL1*	0.6	2.0	3	2	99.5
Q9BX40	protein LSM14 homolog B	*LSM14B*	0.6	8.1	2	2	42.0
Q16222-1	UDP-N-acetylhexosamine pyrophosphorylase	*UAP1*	0.6	6.9	5	3	58.7
Q6WCQ1-2	Isoform 2 of Myosin phosphatase Rho-interacting protein	*MPRIP*	0.6	5.1	4	3	118.0
Q13753-1	Laminin subunit gamma-2	*LAMC2*	0.6	3.5	7	4	130.9
P29034	protein S100-A2	*S100A2*	0.6	27.6	7	5	11.1
O00461	Golgi integral membrane protein 4	*GOLIM4*	0.5	5.2	5	3	81.8
Q9Y696	Chloride intracellular channel protein 4	*CLIC4*	0.5	13.4	5	2	28.8
P19957	Elafin	*PI3*	0.5	20.5	6	2	12.3
O75110	Probable phospholipid-transporting ATPase IIA	*ATP9A*	0.5	1.6	5	2	118.5
P80188	Neutrophil gelatinase-associated lipocalin	*LCN2*	0.5	43.9	46	7	22.6
Q9P265	Disco-interacting protein 2 homolog B	*DIP2B*	0.5	1.7	3	2	171.4
Q9BPX3	Condensin complex subunit 3	*NCAPG*	0.5	5.5	6	4	114.3
P50440-1	glycine amidinotransferase	*GATM*	0.5	7.8	5	3	48.4

## Supplementary Materials

The following are available online at https://www.mdpi.com/2072-6694/11/10/1430/s1, Table S1: List of identified proteins in GEO and GEO-CR CRC cells by quantitative nanoLC MS/MS, Figure S1: In vitro preclinical model of GEO and GEO-CR cells colon cancer cells, Figure S2: Gene ontology biological process and molecular enrichment analysis by the FunRich software on proteins identified in GEO and GEO-CR cell lines by nanoLC-MS/MS, Figure S3: Enrichment analysis by the Innate DB software on proteins identified in GEO and GEO-CR cell lines by nanoLC-MS/MS, Figure S4: MIF inhibition affects human LIM1215-CR and SW48-CR colon cancer cell proliferation, Figure S5: MIF expression by western blot analysis in GEO-CR cells following cetuximab and 4-IPP treatment, Figure S6: Representative profiles and percentages of GEO-CR cell populations in the G0/G1, S, and G2/M phases following cetuximab and 4-IPP treatment, Figure S7: Combined treatment with cetuximab and 4-IPP induce apoptosis in LIM1215-CR and SW48-CR cells, Figure S8: MIF expression by western blot analysis in sensitive GEO cells following cetuximab and hrMIF treatment, Figure S9: Densitometry readings/intensity ratios normalized with tubulin and whole western blots showing all the bands and molecular weight markers of sections reported in Figure 2D, Figure S10: Densitometry readings/intensity ratios normalized with tubulin of western blot sections reported in Figure 5C, Figure S11: Whole western blots showing all the bands and molecular weight markers of sections reported in Figure 5D.
