# Supplementary material for: Macrophage Migration Inhibitory Factor Is a Molecular Determinant of the Anti-EGFR Monoclonal Antibody Cetuximab Resistance in Human Colorectal Cancer Cells
Source: Cancers (Basel). 2019 Sep 25;11(10):1430. doi: 10.3390/cancers11101430 (PMC6826402; doi:10.3390/cancers11101430)
Supplement: Supplementary file 1 [file cancers-11-01430-s001.zip › cancers-580901-suppl-final/cancers-580901-supplementary material.docx]

Supplementary Materials: Macrophage Migration Inhibitory Factor is a Molecular Determinant of the Anti-EGFR Monoclonal Antibody Cetuximab Resistance in Human Colorectal Cancer Cells

Rosita Russo, Nunzia Matrone, Valentina Belli, Davide Ciardiello, Mariangela Valletta, Sabrina Esposito, Paolo Vincenzo Pedone, Fortunato Ciardiello, Teresa Troiani and Angela Chambery

**Table S1.** List of identified proteins in GEO and GEO-CR CRC cells by quantitative nanoLC MS/MS. (In the compressed excel file).

.

**Figure S1.** In vitro preclinical model of GEO and GEO-CR cells colon cancer cells. Sensitive (GEO) and cetuximab-resistant (GEO-CR) cells were treated with increasing concentrations of cetuximab (range: 0.05–20 µg/mL) for 72 h and cell viability were evaluated by MTT assay. Results represent the median of three separate experiments, each performed in triplicate.

**Figure S2.** Functional enrichment based on gene ontology categories on proteins identified in both sensitive and cetuximab-resistant GEO cell lines. (**A**) The enrichment analysis performed for the biological process Gene Ontology category revealed, a significant enrichment of proteins involved in signal transduction, cell communication and cell growth. (**B**) The enrichment analysis for the molecular function category revealed, a significant enrichment of proteins involved in protein serine/threonine kinase activity, RNA, cytoskeletal protein and calcium binding and receptor signalling complex scaffold activity. Enrichment analysis was based on hypergeometric test by the FunRich software. Percentages of mapped proteins/genes and *p*-values are reported on bar graphs along with reference *p*-value (*p* = 0.05).


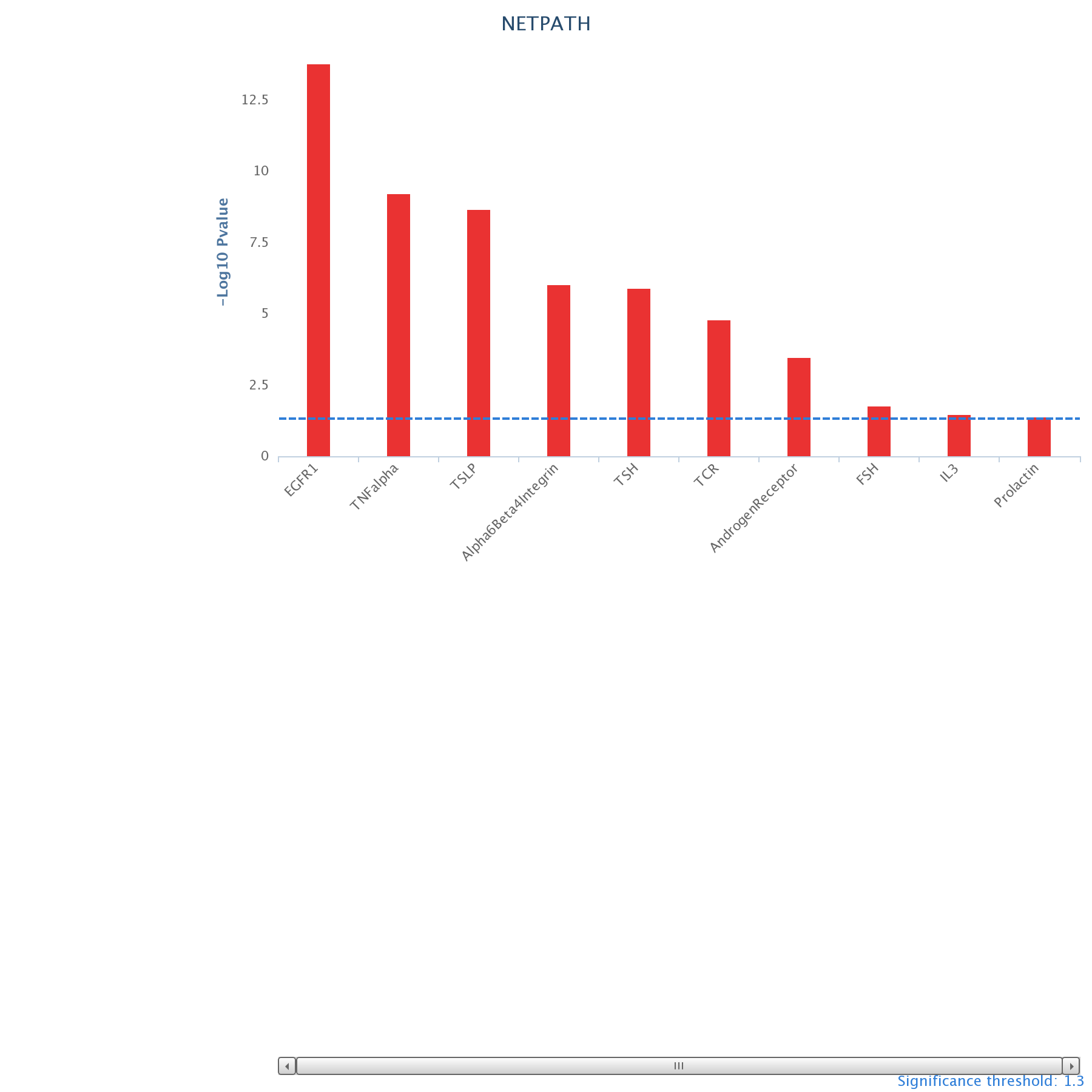


**Figure S3.** Enrichment analysis by the Innate DB software. The enrichment analysis performed on identified proteins in both sensitive and cetuximab-resistant GEO cell lines by nanoLC-MS/MS against the manually curated human cancer and immune signalling pathways “NetPath” revealed a significant enrichment of proteins mapping on EGFR1 pathway. These proteins have been mapped on the protein network reported in Figure 1B.


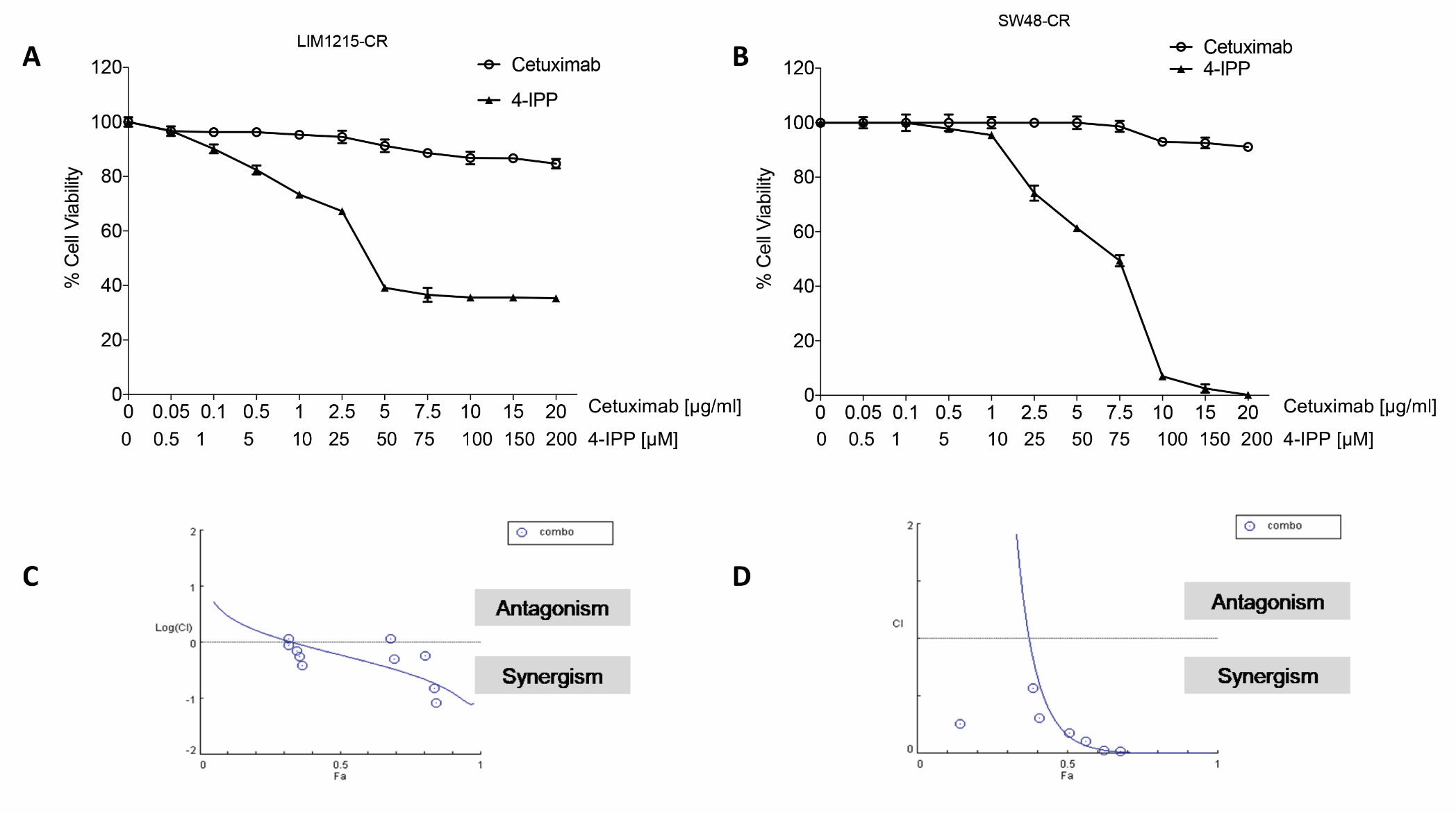


**Figure S4.** MIF inhibition affects human colon cancer cell proliferation. (**A**,**B**) Cell proliferation was evaluated by MTT staining on LIM1215-CR (**A**) and SW48-CR (**B**) cells following treatment for 72 h with cetuximab (range: 0.05–20 µg/mL) and 4-IPP (range: 0.5–200 µM). Results represent the median of three separate experiments, each performed in triplicate. (**C**,**D**) Combination Index (CI) values for combined treatments with cetuximab plus 4-IPP on LIM1215-CR (**C**) and SW48-CR (**D**); *bars*, standard deviation (SD).

**Figure S5.** Western blot analysis showing the expression of MIF in GEO-CR cell lines following treatment with cetuximab (2.5 μg/mL) and 4-IPP (25 μM) for 72 h as single agents and in combination. The monoclonal anti-α-tubulin antibody was used as loading control antibody.

**Figure S6.** (**A**) Representative profiles of cell cycle distribution in of GEO-CR cells treated with cetuximab (2.5 µg/mL), 4-IPP alone (25 µM) and their combination for 24 h. (**B**) Percentages of cell populations in the G0/G1, S, and G2/M phases.

**Figure S7.** Combined treatment with cetuximab and 4-IPP induces apoptosis in LIM1215-CR and SW48-CR cells. Apoptosis assay on LIM1215-CR and SW48-CR cells following treatment with cetuximab (2.5 µg/mL), 4-IPP (25 µM) and their combination for 24 h. The graph bar shows the apoptotic cell percentage of three independent experiments; bars, standard deviation (SD); combination versus 4-IPP single treatment ** *p* < 0.01.

**Figure S8.** Western blot analysis showing the expression of MIF in cetuximab-sensitive GEO cells following treatment with cetuximab (2.5 μg/mL) and human recombinant MIF (hrMIF, 100 ng/mL) for 72 h as single agents and in combination. The monoclonal anti-α-tubulin antibody was used as loading control antibody.


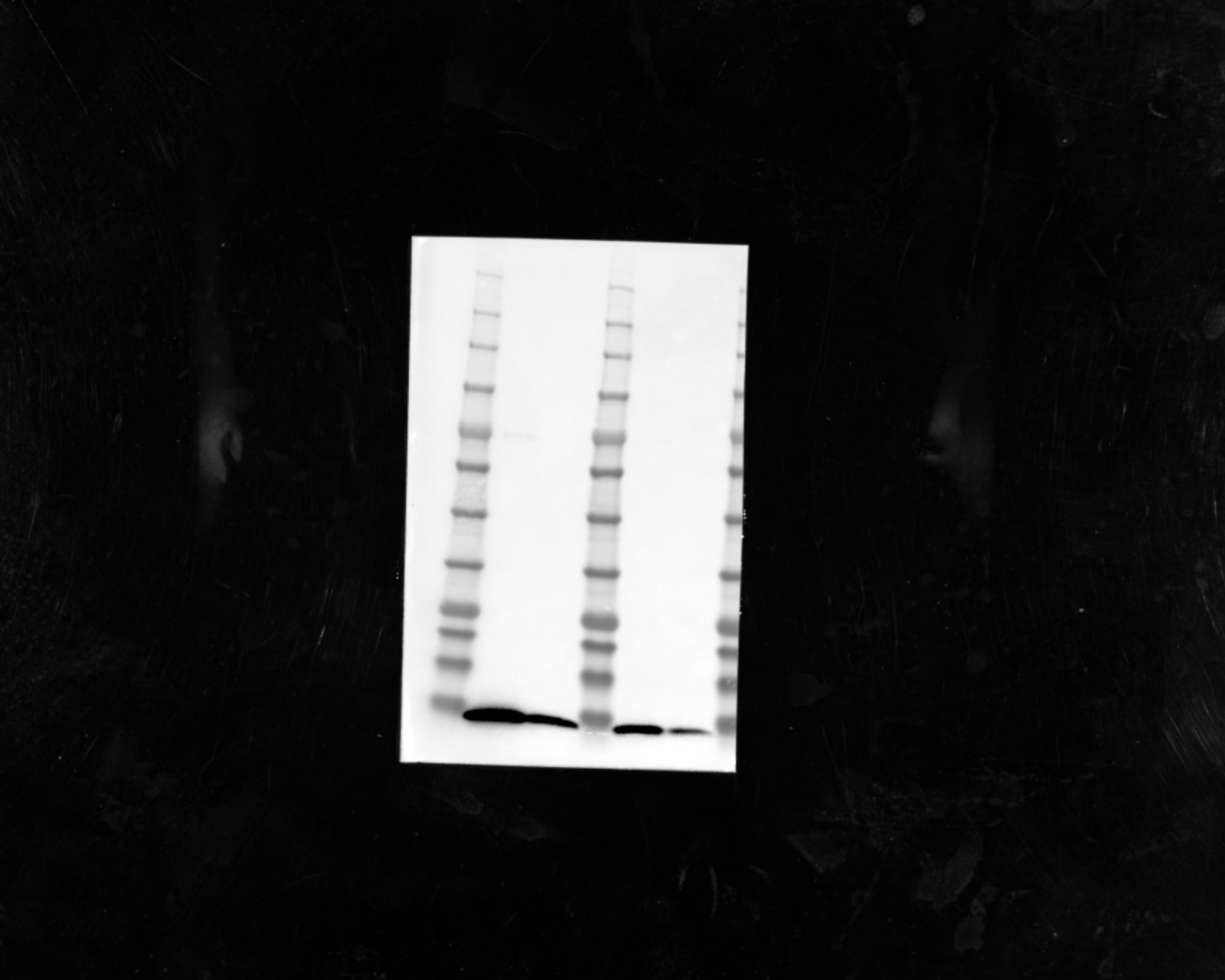


- 245 kDa

- 180 kDa

- 135 kDa

- 100 kDa

- 75 kDa

- 63 kDa

- 48 kDa

- 35 kDa

- 25 kDa

- 20 kDa

- 17 kDa

- 11 kDa

MIF (~12 kDa)


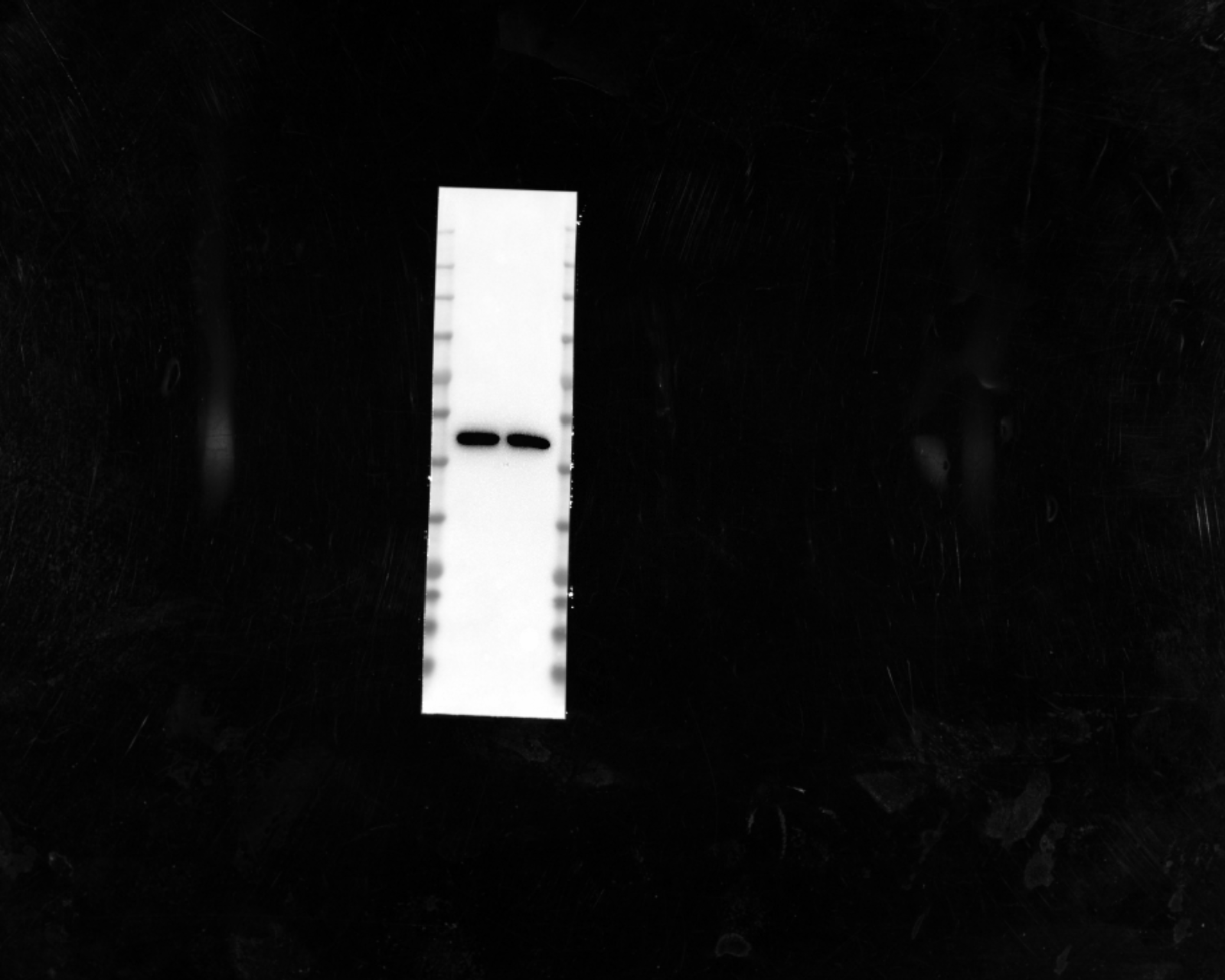


Tubulin (~50 kDa)


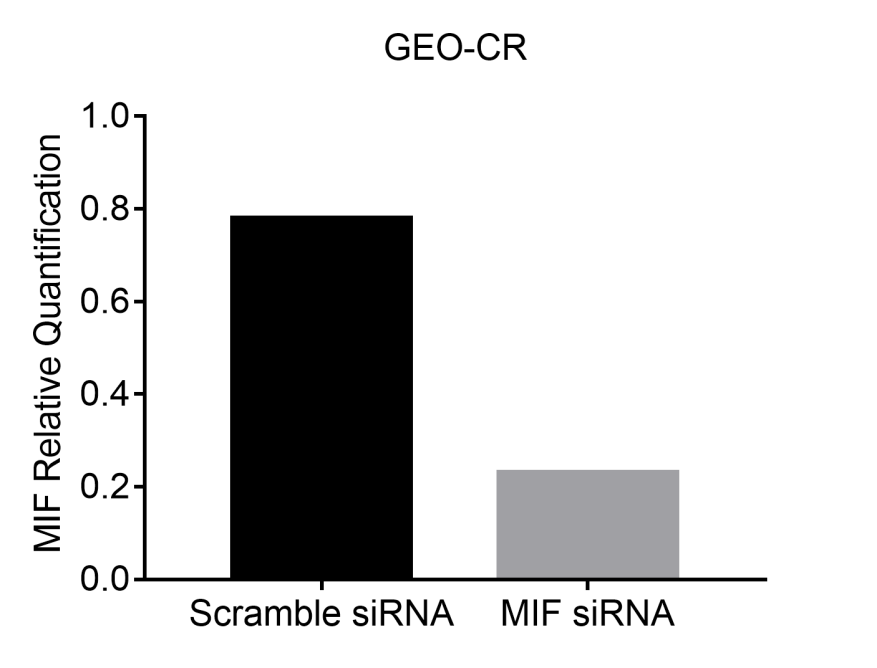


**Figure S9.** Densitometry readings/intensity ratios normalized with tubulin and whole western blots showing all the bands and molecular weight markers of sections reported in Figure 2D.


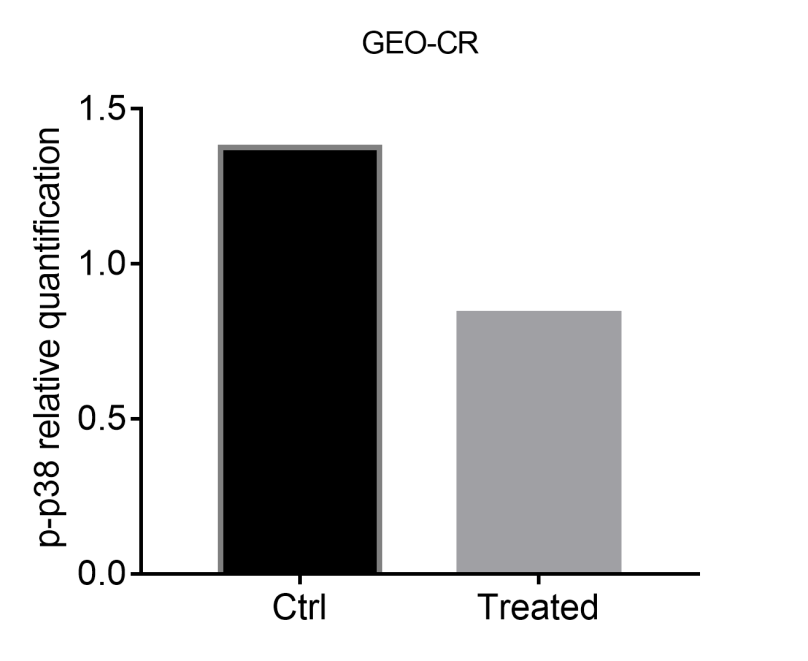

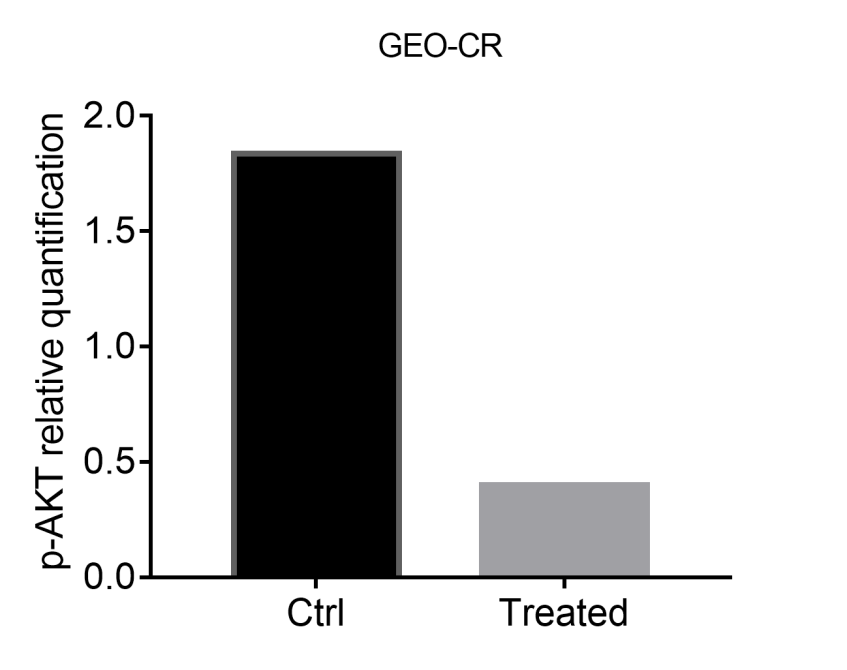

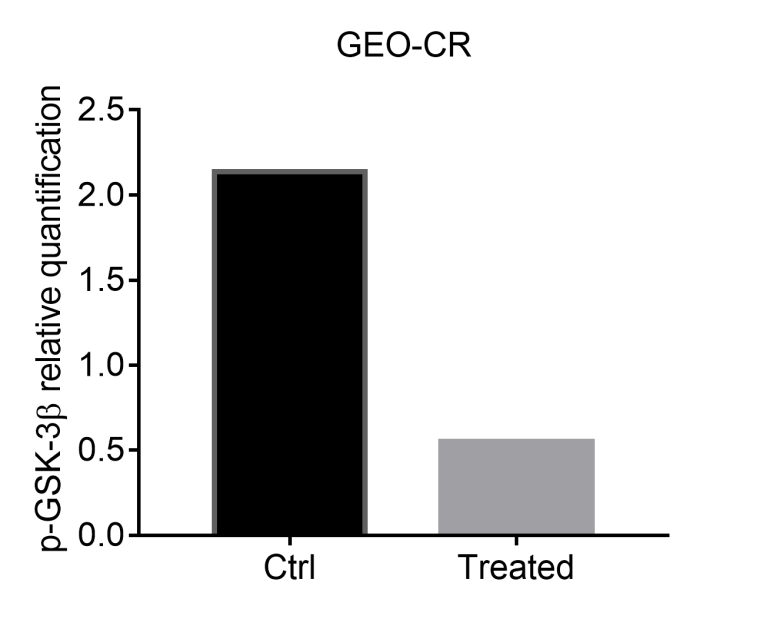

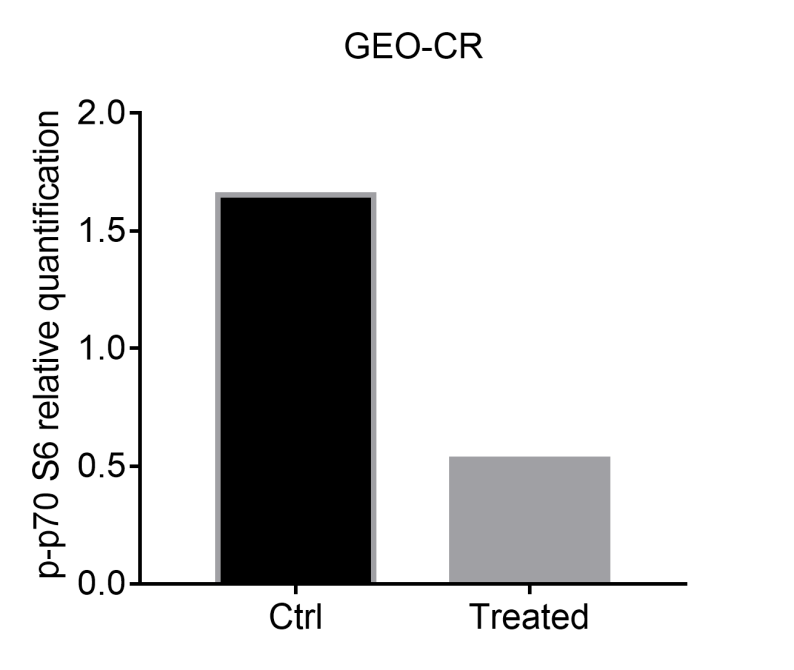

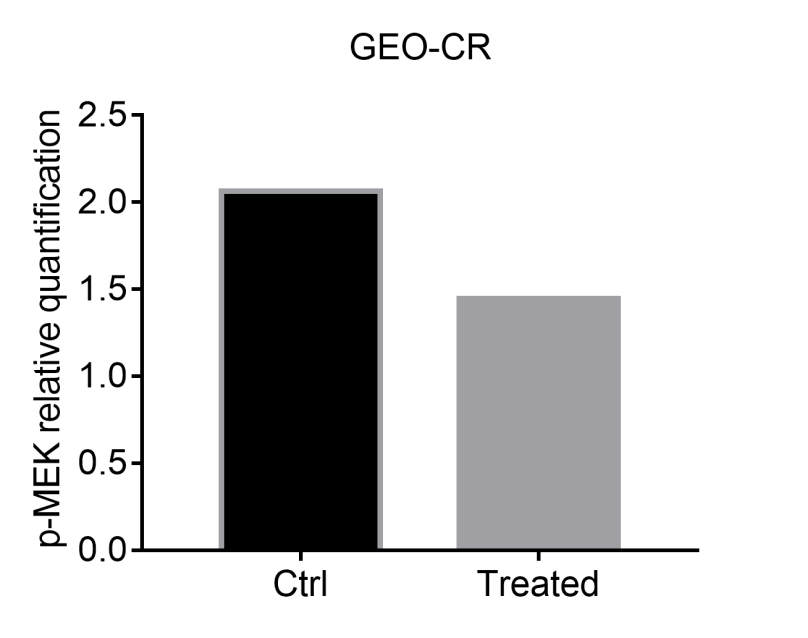


**Figure S10.** Densitometry readings/intensity ratios normalized with tubulin of western blot sections reported in Figure 5C.

C

T

C

T

C

T

C

T

C

T

C

T

**Figure S11.** Whole western blots showing all the bands and molecular weight markers of sections reported in Figure 5D.

| 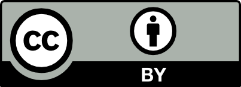 | © 2019 by the authors. Licensee MDPI, Basel, Switzerland. This article is an open access article distributed under the terms and conditions of the Creative Commons Attribution (CC BY) license (http://creativecommons.org/licenses/by/4.0/). |
| --- | --- |
